# *Yersinia enterocolitica* Bacteremia Associated with a Ruptured Abdominal Aortic Aneurysm: A Case Report with Literature Review

**DOI:** 10.3390/microorganisms11122911

**Published:** 2023-12-02

**Authors:** Yordan Kalchev, Hristina Urdzhanova, Stefan Stanev, Bogomila Cheshmedzhieva, Maria Pavlova, Gergana Lengerova, Marianna Murdjeva

**Affiliations:** 1 Department of Medical Microbiology and Immunology “Prof. Dr. Elissay Yanev”, Faculty of Pharmacy, Medical University of Plovdiv, 4002 Plovdiv, Bulgaria; 2 Laboratory of Microbiology, University Hospital St. George, 4000 Plovdiv, Bulgaria; 3 Research Institute, Medical University of Plovdiv, 4002 Plovdiv, Bulgaria; 4 Department of Pathology, Zealand University Hospital, 4000 Roskilde, Denmark; 5 Clinic of Vascular Surgery, University Hospital St. George, 4000 Plovdiv, Bulgaria; 6 National Reference Laboratory of Enteric Infections, Pathogenic Cocci and Diphtheria, Department of Microbiology, National Center of Infectious and Parasitic Diseases, 1504 Sofia, Bulgaria; mimipavlovaa@gmail.com

**Keywords:** abdominal aortic aneurysm, bacteremia, MALDI-TOF, vascular disease, *Yersinia enterocolitica*, *Yersinia* spp., PCR, ruptured aneurysm, cardiovascular disease, aorta

## Abstract

*Yersinia enterocolitica* is a foodborne pathogen, mainly associated with disorders involving the gastrointestinal tract, including diarrhea, ileitis, and mesenteric lymphadenitis. Extraintestinal presentation is uncommon in healthy individuals, but bacteremia is reported in immunocompromised hosts. We present a 74-year-old male with *Y. enterocolitica* serogroup O:3 bacteremia who complicated to rupture of an abdominal aortic aneurysm. With the current case report, we aimed to emphasize the association of *Y. enterocolitica* bacteremia with abdominal aortic aneurysm rupture. Better surveillance is needed, not only to reduce morbidity and mortality but also to update current epidemiological data on the incidence of such associations.

## 1. Introduction

*Yersinia enterocolitica* is a foodborne pathogen, mainly associated with gastrointestinal tract disorders, including diarrhea, ileitis, mesenteric lymphadenitis, and intussusception [1,2,3]. However, it is a relatively uncommon cause of diarrhea and abdominal pain [1,4]. Pigs are the main reservoir for the bacterium and, thus, intestinal yersinosis is mainly caused by the consumption of contaminated raw or undercooked pork meat [1]. According to CDC data, *Y. enterocolitica* is responsible for approximately 117,000 illnesses, 640 hospitalizations, and 35 deaths in the United States every year [4]. Extraintestinal presentation is uncommon in healthy individuals, but bacteremia is reported in immunocompromised hosts, with mortality up to 50% [1,4,5].

Increasing attention is paid to the role of the bacterium in a variety of clinical entities, such as Crohn’s disease, incomplete Kawasaki diseases, myopericarditis, septic arthritis, as well as primary cellulitis and skin abscess [6,7,8,9,10,11,12]. With the current case report, we aimed to emphasize the association of *Y. enterocolitica* bacteremia with ruptured abdominal aortic aneurysm (AAA), to contribute to collecting new epidemiological data, and to perform a literature review of the reported clinical cases.

## 2. Case Description

A 74-year-old male was admitted to the Clinic of Vascular Surgery of University Hospital St. George, Plovdiv, Bulgaria. At admission, the patient presented with abdominal pain, increased heart rate (up to 110 beats per minute), elevated blood pressure (160/100 mmHg), and pale skin. The patient was awake, alert, and oriented. A pulsating mass was detected on palpation of the abdomen. Peripheral pulses were preserved. Comorbidities were as follows: arterial hypertension, chronic obstructive pulmonary disease, chronic kidney disease, smoking, moderate alcohol abuse, and no history of I.V. or other drug use. The assigned antihypertensive therapy by the general practitioner included bisoprolol 5 mg once daily and olmesartan/hydrochlorothiazide 20 mg/12.5 mg once daily.

Blood testing demonstrated decreased hemoglobin levels to 112 g/L (reference for males 140–180 g/L), increased white blood cell (WBC) count of 19.91 × 10^9^/L (reference 3.5–10.5 × 10^9^/L), with 90.9% neutrophil predominance, increased C-reactive protein (CRP) of 349 mg/L (reference below 0–10 mg/L), and accelerated erythrocyte sedimentation rate (ESR) of 76 mm/h (reference < 20 mm/h for males over 50 years of age). 

**Figure 1:**
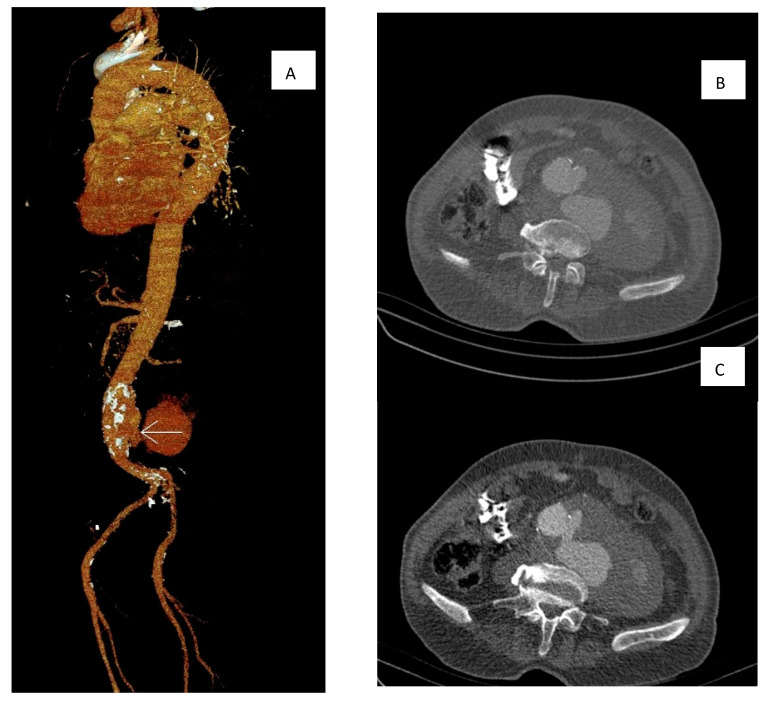
Multidetector Computer tomography angiography was performed showing a ruptured distal abdominal aorta. (**A**) Reconstruction of the MDCTA showing the jet (arrow) and retroperitoneal hematoma. (**B**) Jet from the ruptured aneurysm. (**C**) Maximum hematoma size.

Multidetector computer tomography angiography (MDCTA) was performed, showing a ruptured distal abdominal aorta with left-sided retroperitoneal leakage, with no evidence of aorto-caval fistula (Figure 1). Empirical antibiotic therapy with ceftriaxone was initiated. The patient underwent an emergency vascular repair. The retroperitoneal space was reached through a median laparotomy. The aneurysm was clamped under the renal arteries. The aneurysmal sack was opened with a longitudinal incision. After the removal of the mural thrombus, an aorto-caval fistula was verified, and it was sutured with prolene 4–0. Aneurysm resection and silver-knitted dacron tube graft interposition with end-to-end anastomoses were performed. Intraoperative blood loss was approximately 0.5 L. Total perioperative blood transfusion was approximately 1.2 L red blood cell transfusion and 910 mL plasma transfusion. 

An intraoperative blood sample was collected for aerobic and anaerobic blood culturing. After 2 days of incubation using BacT/ALERT (bioMérieux, Marcy-l’Étoile, France), the blood culture became positive. Gram staining revealed Gram-negative rods. After an overnight incubation on eosine-methylene blue agar, small lactose-negative round colonies appeared. The time to isolate identification was reduced by applying matrix-assisted laser desorption/ionization–time-of-flight mass spectrometry—MALDI-TOF MS (Vitek MS, bioMérieux, Marcy-l’Étoile, France). Vitek-2 Compact (bioMérieux, Marcy-l’Étoile, France) was also used for identification and antimicrobial susceptibility testing. Both methods identified the isolate as *Y. enterocolitica*. The results of postoperative blood and urine cultures were negative.

The antimicrobial susceptibility testing was performed on Vitek-2 Compact by using the antimicrobial card AST-N 204. The isolate was susceptible to ampicillin, amoxicillin/clavulanic acid, piperacillin/tazobactam, cefotaxime, ceftazidime, cefepime, ertapenem, imipenem, meropenem, amikacin, gentamicin, ciprofloxacin, and trimethoprim/sulfamethoxazole (Table 1). Antimicrobial susceptibility was interpreted according to the EUCAST breakpoints.

Molecular confirmation and biotyping methods were performed in the National Reference Laboratory of Enteric Infections, Pathogenic Cocci, and Diphtheria at the National Center of Infectious and Parasitic Diseases (NCIPD), Sofia. The clinical isolate was confirmed as *Y. enterocolitica* by PCR-based assays for the detection of specific virulence genes *ail* (a chromosomal attachment invasion gene in pathogenic strains only), *ystA* (a plasmid-borne virulence gene responsible for the production of a heat-stable enterotoxin), and *ystB* (a gene encoding an enterotoxin of *Y. enterocolitica*) [13]. Phenotypic serotyping of the strain was performed by anti-*Y. enterocolitica* sera (Sifin, Germany) and serotyped it as *Y. enterocolitica* O:3.

Following a post-operative recovery period, the patient was discharged from the hospital on postoperative day 7 with improved laboratory parameters and clinical condition. He was treated with 6 weeks of intravenous ceftriaxone. The patient was seen in the clinic ∼6 weeks after the treatment, with no complaints, and a subsequent CT scan did not demonstrate any evidence of residual or recurrent infection. 

Written informed consent was obtained from the patient.

## 3. Discussion

There are at least 18 different species identified in the genus of *Yersinia*. The majority are environmental bacteria that can be found in soil and aquatic areas. Three of them are known to cause infections in humans, including the renowned *Y. pestis* (the causative agent of bubonic plague), *Y. pseudotuberculosis*, and *Y. enterocolitica*. Currently, among *Yersinia* spp., *Y. enterocolitica* is the predominant bacterium being recovered from human specimens, including both pathogenic and non-pathogenic strains [14,15,16,17,18]. 

In healthy individuals, the immune response is sufficient to clear the *Y. enterocolitica* gastrointestinal infection, so the disease is usually presented as self-limiting infectious diarrhea. However, in immunocompromised persons such as the elderly, reduced local immunity could be associated with the translocation of the bacteria from the gut into the bloodstream, leading to bacteremia, which is uncommon in immunocompetent individuals. Comorbidities that are known to contribute to *Y. enterocolitica* bacteremia include diabetes, liver cirrhosis, malignant diseases, and iron overload [19,20]. The presented patient did not have any of these comorbidities but had chronic obstructive pulmonary disease due to excessive smoking and arterial hypertension. Smoking is a known factor, causing AAA development and increasing rupture in at-risk populations [21]. In addition, epidemiological studies have shown an inverse association between diabetes mellitus and the incidence of AAA. However, diabetes mellitus was absent in the presented patient [22]. Thus, we can conclude that the progressive decline in immune function with increasing age or immunosenescence could have been the most important predisposing factor for developing bacteremia in the presented patient. Nevertheless, it should be noted that the sex of the patient and the presence of arterial hypertension are known as independent risk factors for the development of AAA itself [19,20].

AAA represents a dilatation of the abdominal aortic wall that is permanent and exceeds 3 cm. The data show that the incidence of AAA is steadily increasing, mainly attributed to the aging population and increased detection rates [23,24]. The pathogenesis is complex and includes inherited and environmental risk factors [20,25]. The major problem with AAAs is that they are usually asymptomatic until rupture, leading to more than 80% mortality overall. If clinical symptoms are present, they are usually non-specific [24,26,27]. Another problem is that AAAs can become infected, leading to degradation of the aneurysmal wall, thus significantly increasing the risk of rupture and complicating the treatment. Although these situations are rare and predominantly seen in immunocompromised hosts, the mortality rate can be up to 44% [26,27,28,29]. Patients who experience systemic inflammatory response syndrome are at increased risk for poor surgical outcomes [30]. Early recognition and emergency surgical repair are essential to improve survival rates. Most patients with AAA infection show increased markers of inflammation, such as WBC, CRP, and ESR, all of which were elevated in our patient. Blood cultures usually yield a positive result between 50 and 75% [31,32]. We also managed to successfully recover the bacterium from an intraoperative blood culture. 

The term mycotic aneurysm was introduced by Osler to describe the aneurysms that are seen in patients with infective endocarditis. Regardless of the misleading term, these are primarily due to bacterial pathogens, and fungal involvement is rare but possible [33]. Currently, many authors use the term mainly to refer to any kind of infected aneurysm, regardless of the pathogenesis [27,34]. 

The leading pathogens infecting atherosclerotic aneurysms are *Staphylococcus aureus*, non-hemolytic *Streptococcus* spp., *Salmonella enterica*, Gram(−)negative enteric bacteria such as *E. coli*, *Bacteroides* spp., etc. Vascular infections attributed to *Yersinia* spp. are considered rare and mainly due to *Y. enterocolitica,* with few case studies recognizing *Y. pseudotuberculosis* as a causative agent [27,30,31,35,36,37,38,39].

As of July 2023, we were able to find 16 reports in the literature, including a total of 23 patients of *Y. enterocolitica* associated with aneurysms (Table 2). Based on the reported age of the patients in the literature, we can conclude that the disease is exclusively presented in elderly men over 50 years old with a median age of 70 years old (excluding data from Mercié P et al., where the precise age of the three reported patients cannot be extracted). The case-fatality rate was calculated to be 39%.

Gastrointestinal symptoms are reported in just 35%; hence, their absence should not rule out the possibility of *Y. enterocolitica* association. Probably, a medical history of recent gastroenteritis, not presenting some days before the time of admission, could be of use to draw attention to *Y. enterocolitica* infection. It is worth mentioning, however, that some of the gastrointestinal symptoms can also be explained by the aneurysmal growth itself.

The literature review confirms that blood cultures are the optimal clinical specimen to recover *Y. enterocolitica* in these patients. In addition, it is reported that the bacterium can cause infective endocarditis [56]. Given the emergency, transoesophageal echocardiography was not performed to rule out concomitant endocarditis in the presented case report.

Serogroups O:3; O:5,27; O:8; and O:9 of *Y. enterocolitica* are commonly reported in human infection [1,4]. The case reports of *Y. enterocolitica* in aneurysms identified serogroups O:3 and O:9 as predominant. These data correlate with the phenotypically and molecularly confirmed clinical isolate of this case report. According to the PCR analysis, the clinical strain was determined as biotype 1B *Y. enterocolitica* strain, because of the presence of virulent genes *ail*, *ystA*, and lack of the *ystB* gene. In humans, 1B is the predominant genotype of *Y. enterocolitica* as well as strains of serotype O:3, whereas animal isolates of *Y. enterocolitica* belong predominantly to biotype 4 [57,58].

It is worth mentioning that based on the antimicrobial susceptibility report that showed the isolate was susceptible to all tested antibiotics, the recovered *Y. enterocolitica* could be considered a wild-type isolate and not a healthcare-associated pathogen. 

*Y. enterocolitica* is part of the group of enteropathogenic bacteria—*Salmonella* spp., *Shigella* spp., Diarrheagenic *E. coli* (DEC), and *Campylobacter* spp.—which are routinely examined in microbiological laboratories in Bulgaria in the diagnosis of enterocolitis as well as prophylactic examinations of close contacts. *Y. enterocolitica* has the lowest frequency of isolation from human specimens in Bulgaria. Data from the NCIPD show an average of 10–15 registered cases per year in the country. Epidemiological studies conducted at the National Reference Laboratory of Enteric Infections, Pathogenic Cocci and Diphtheria at the NCIPD confirm the consumption of home-made, raw pork sausages as the main source of gastrointestinal infections caused by *Y. enterocolitica* and *Y. pseudotuberculosis*. In the last 10 years, no epidemic of *Y. enterocolitica* has been registered on the territory of the Republic of Bulgaria, unlike other EU member countries, with Germany and France leading in the number of reported yersinioses for 2020 and 2021, according to the European Centre for Disease Prevention and Control data [59].

The conventional microbiological diagnosis of *Y. enterocolitica* might be challenging, and successful isolation and subsequent identification require specific selective media and tests to be available in the laboratory. Currently, MALDI-TOF mass spectrometry testing is considered a rapid, accurate, and inexpensive alternative to conventional approaches for the identification of *Y. enterocolitica*. The method, where implemented, can replace the old and time-consuming biochemical and phenotypical techniques for the identification of the bacterium in the microbiological laboratory.

Being a foodborne pathogen, *Y. enterocolitica* infections could be limited by avoiding eating undercooked or raw meat, drinking unpasteurized milk, or eating related dairy, and proper hand washing. High-risk patients, such as those with vascular diseases, could be actively educated, thus limiting the incidence of such life-threatening complications. In addition, the bacterium should be recognized as a potential trigger of aneurysmal rupture and should be considered by physicians in the differential diagnosis of specific populations with cardiovascular diseases. In addition, physicians should notify the laboratory when a high index of suspicion is present.

## 4. Conclusions

The presented case report expands the limited epidemiological data on the significance of *Y. enterocolitica* bacteremia in patients with cardiovascular diseases. *Y. enterocolitica* could be considered as a potential agent that, during bacteremia, could lead to the rupture of an AAA. Implementing routine screening of *Y. enterocolitica*, especially in elderly male patients who are smokers, could possibly contribute to improved surveillance, collect further epidemiological data, and expand our knowledge on such associations. 

## Figures and Tables

**Table 1:** Antimicrobial susceptibility based on Vitek-2 Compact AST-N204.

Antibiotic	MIC * Value	Interpretation
ampicillin	≤4	S
amoxicillin/clavulanic acid	≤4	S
piperacillin/tazobactam	≤4	S
cefotaxime	≤1	S
ceftazidime	≤1	S
cefepime	≤1	S
ertapenem	≤0.5	S
imipenem	≤0.25	S
meropenem	≤0.25	S
amikacin	≤4	S
gentamicin	≤1	S
ciprofloxacin	≤0.25	S
trimethoprim/sulfamethoxazole	≤20	S

* MIC—minimum inhibitory concentration.

**Table 2:** Case reports on *Y. enterocolitica* associated with aneurysms.

№	Year	Age	Sex	First Isolated from	GI Symptoms	Site of Aneurysm	Serogroup/ Biogroup	Outcome	Ref.
1.	1981	53	M	Stool and blood	Yes	Carotid artery	Biotype 4	Recovered	[40]
2.	1985	79	M	Blood	Yes	AAA	Serotype O:3, Biotype 4	Fatal	[41]
3.	1987	76	M	Aneurysmal wall	Yes	AAA	Serotype O:9, Biotype 2	Recovered	[42]
4.	1993	59	M	Blood	Yes	AAA	Biotype 4	Fatal	[43]
5. 6 cases	1993	77, 55, 76, 75, 84, 68	M	Blood (4) unspecified (2)	No	AAA (4) Popliteal Artery (2)	Serotype O:9 (4) unspecified (2)	Fatal (2/6)	[44]
6.	1994	57	M	Resected aorta	No	AAA	Serotype O:3, Biotype 4	Fatal	[45]
7. 3 cases	1996	>70	M	Blood	No	AAA	Serotype O:9	Fatal (2/3)	[46]
8.	1996	64	M	Blood	Yes	Femoral artery and AAA	not reported	Fatal	[47]
9.	1997	78	M	Blood	No	AAA	Serotype O:9, Biotype 2	Recovered	[48]
10.	1998	55	M	Blood	No	AAA	Serotype O:3, Biotype 4	Fatal	[49]
11.	2004	70	M	Vascular graft	No	AAA	Serotype O:3, Biotype 4	Recovered	[50]
12.	2014	74	M	Blood	Yes	AAA	Biotype 2	Recovered	[51]
13.	2014	78	M	Blood	No	AAA	not reported	Recovered	[52]
14.	2015	68	M	Blood	No	AAA	not reported	Recovered	[53]
15.	2017	72	M	Blood	Yes	AAA	not reported	Recovered	[54]
16.	2018	63	M	Blood and aneurysmatic aortic wall	Yes	AAA	Serotype O:9, Biotype 2	Recovered	[55]

GI—gastrointestinal; Ref.-reference; AAA—abdominal aortic aneurysm.

## Data Availability

Data are available from the corresponding author on a reasonable request.
